# Economic Returns to Investment in AIDS Treatment in Low and Middle Income Countries

**DOI:** 10.1371/journal.pone.0025310

**Published:** 2011-10-05

**Authors:** Stephen Resch, Eline Korenromp, John Stover, Matthew Blakley, Carleigh Krubiner, Kira Thorien, Robert Hecht, Rifat Atun

**Affiliations:** 1 Harvard School of Public Health, Center for Health Decision Science, Boston, Massachusetts, United States of America; 2 The Global Fund to Fight AIDS, Tuberculosis and Malaria, Geneva, Switzerland; 3 Department of Public Health, University Medical Center, Rotterdam, The Netherlands; 4 Futures Institute, Glastonbury, Connecticut, United States of America; 5 Results for Development Institute, Washington, District of Columbia, United States of America; 6 Imperial College, London, United Kingdom; Boston University, United States of America

## Abstract

Since the early 2000s, aid organizations and developing country governments have invested heavily in AIDS treatment. By 2010, more than five million people began receiving antiretroviral therapy (ART) – yet each year, 2.7 million people are becoming newly infected and another two million are dying without ever having received treatment. As the need for treatment grows without commensurate increase in the amount of available resources, it is critical to assess the health and economic gains being realized from increasingly large investments in ART. This study estimates total program costs and compares them with selected economic benefits of ART, for the current cohort of patients whose treatment is cofinanced by the Global Fund to Fight AIDS, Tuberculosis and Malaria. At end 2011, 3.5 million patients in low and middle income countries will be receiving ART through treatment programs cofinanced by the Global Fund. Using 2009 ART prices and program costs, we estimate that the discounted resource needs required for maintaining this cohort are $14.2 billion for the period 2011–2020. This investment is expected to save 18.5 million life-years and return $12 to $34 billion through increased labor productivity, averted orphan care, and deferred medical treatment for opportunistic infections and end-of-life care. Under alternative assumptions regarding the labor productivity effects of HIV infection, AIDS disease, and ART, the monetary benefits range from 81 percent to 287 percent of program costs over the same period. These results suggest that, in addition to the large health gains generated, the economic benefits of treatment will substantially offset, and likely exceed, program costs within 10 years of investment.

## Introduction

Flows of bilateral and multilateral aid over the past decade, combined with the domestic financial contributions of many countries, have fueled a remarkable scale-up in AIDS treatment and prevention programs in low and middle income countries. Starting with just a few thousand patients in 2002, UNAIDS and WHO report that by the end of 2009 more than five million people were enrolled in antiretroviral therapy (ART) programs in these countries [1]. Despite these impressive gains, only a third of the estimated 15 million HIV-infected persons with the most acute need (according to the WHO's 2010 eligibility definition) have access to treatment [1], [2]. Each year two million people still die from AIDS (most without having ever received ART) and approximately 2.7 million persons are newly infected by HIV [3], [4].

Several studies have suggested that the intrinsic value of the health gains generated from ART is worth the cost of treatment, thereby arguing for greater investment in ART programs to meet growing treatment needs [5], [6]. However, the 2008–2010 global recession, flattening aid budgets, and fiscal tightening in many AIDS-affected countries are threatening the ability of donors and countries to continue scaling up ART. In this context, policy makers deciding whether to commit additional resources to ART programs will want to consider not only the cost and health impacts of program continuation, but also the likely economic benefits of doing so.

To estimate the societal-level economic impact of ART, we analyzed three streams of benefits from AIDS treatment accruing over time to a cohort of patients enrolled on treatment in programs supported by the Global Fund: (1) restored labor productivity amongst workers with AIDS, (2) orphan care expenditures avoided because parents remain alive on ART, and (3) delayed end-of-life care costs associated with death from AIDS. These streams of economic benefits were selected because they offset the cost of treatment over short time horizons and therefore may be especially salient to policy-makers concerned with health budgets, household economic stability and societal-level economic growth. Our model is applied to patients in AIDS programs across low and middle income countries where the Global Fund to Fight AIDS, Tuberculosis and Malaria (Global Fund) cofinances ART, alongside domestic and other external funding. Results should be seen as broadly applicable to national ART programs financed by other international organizations and from domestic public and private sources.

## Materials and Methods

### Overview

The full cohort of 3.5 million ART patients who participate in treatment programs co-financed by the Global Fund are found across 98 countries (Table S1), 80 percent of these in just 20 African countries. The health impact and gross program cost of maintaining this cohort of patients over the next decade were estimated using methods and data detailed in a companion paper [7]. We extend the model, in order to estimate economic returns from this investment. Our approach to estimating the economic returns of the ART program is to compare it to a ‘null scenario’ in which such a treatment effort does not exist [8], [9]. We thus calculated the ratio of the full program costs (of which a portion is financed by Global Fund) to the set of economic benefits generated through the program. In doing so, we estimated the return on investment of the ART program effort. A summary of model parameters is shown in Table 1.

**Table 1:** Key model parameters.

Parameter	Base Case (source)	Sensitivity analysis
Patients alive on ART in 2011	Patient targets of Global Fund-supported ART programs for end 2011 (country-level) [7], [18]	N/A
Survival with and without ART	79.5% survival at 12 months, and 96% for each subsequent year, for all countries [3], [7]	N/A
Value of full-time employment of asymptomatic HIV-infected adults	Gross national income per working age person (GNIpwap) [39], [45]	50% GNIpwap
Labor productivity of untreated symptomatic HIV/AIDS cases relative to asymptomatic HIV-infected adult	20% (see Information S1)	0%, 40%
Labor productivity of patients established on ART relative to asymptomatic HIV-infected adult	75% (see Information S1)	60%, 90%
Fraction of HIV patients that are working age	90% [4]	
Months after starting ART before productivity rebounds	6 (see Information S1)	N/A
Months of reduced productivity associated with treatment failure under ART	12 months before death	N/A
Orphan-years averted per patient-year of ART	Country-specific, varying from 0.32 to 0.76 (average 0.5), computed with *Spectrum* [14] among the 14 countries with largest numbers of Global Fund-supported ART and services for orphans and vulnerable children. Other countries extrapolated adjusting for total fertility rate. Orphans are defined as children under 18 years losing one or both parents	N/A
Fraction of orphans needing care and support	Equal to fraction of full population below nationally defined poverty line, which ranged between 25% and 75% (average across Global Fund-supported ART patients: 46%) in the 14 countries with largest numbers of Global Fund-supported ART and support for orphans and vulnerable children [18]	N/A
Cost of care for orphans and vulnerable children per orphan-year	$224, based on data from 300 NGOs operating in 7400 sites in sub-Saharan Africa, adjusted for expected economies of scale during program scale-up [55]	N/A
End-of-life care of AIDS patients: lifetime cost	$480 in patients without ART [7], [57], $160 in patients with ART, based on an assumed three-fold shorter terminal illness compared to patients not accessing ART	

### Health impact of ART

**Figure 1:**
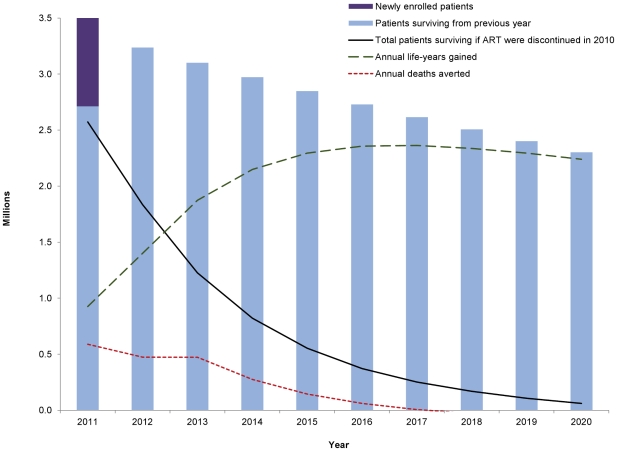
Projected health impact of ARV treatment in Global Fund countries. Survival of newly enrolled and surviving HIV/AIDS patients on ART in Global Fund-supported programs, according to end-2009 grant results and 2010–11 targets of ongoing grants and approved proposals through the 10^th^ round of applications, assuming no additional patient enrolments after 2011. Life years gained is calculated as difference between the ART scenario and a no-ART counterfactual. See [7] for more details.

We first modeled the survival through 2020 of the 3.5 million patients who will be on ART as of 2011 in Global Fund-supported country programs, based on service delivery targets [10], [11], [12] of ongoing Global Fund grants and proposals through the 10^th^ round of applications in December 2010 [13]. To calculate the survival gain attributable to ART, we compared survival on ART to that of untreated patients whose disease has progressed to the point of ART eligibility (e.g. CD4<200 cells/mm3), using established epidemiological methods [14], [15]. We assumed the probability that ART patients survive to be 79.5 percent in their first year and 96 percent for subsequent years [3]. The difference represents the survival benefit (life-years gained) attributable to ART, as illustrated in Figure 1.

### Costs of ART programs

Program-level recurrent costs (of which the Global Fund finances a portion alongside domestic and other donor resources) were estimated by summing the cost of antiretroviral drugs (ARVs), lab`oratory testing and service delivery (personnel, supplies, facilities, etc.). All patients in the cohort have already initiated ART care, so counseling and testing and costs associated with treatment initiation were not included. Country-reported ARV utilization patterns and procurement prices [16], [17] were analyzed to estimate country-specific costs for first-line and second-line ARV regimens. Across the countries in our study, the median annual cost per patient was $204 and $1,238, respectively (see [7]). The median annual cost of monitoring patients with laboratory tests ($180) obtained from a synthesis of 15 published reports from low and middle income countries was applied to the entire cohort [7]). Estimates of service delivery costs were based on eight studies of inpatient and outpatient care for ART patients [7]. The median rates of utilization obtained from these studies were applied to all patients in our analysis. Unit costs per day of hospitalization and per outpatient clinic visit were country-specific, based on WHO-CHOICE estimates [18], [19] and resulted in median service delivery costs of $138 per patient-year [7]. Proportions of patients on each regimen were projected using trends in migration from first- to second-line regimens estimated from treatment data reported by national AIDS programs to the WHO in 2009 [20]. In conducting sensitivity analysis, we considered possible changes in ARV cost and service delivery over the coming decade.

### Benefits: restored labor productivity

AIDS has a substantial negative effect on patients' productivity, since many of those who become infected are of prime working age [21], [22], [23]. As HIV progresses, patients develop opportunistic infections and other symptoms that limit their ability to work. By the time HIV-infected workers are diagnosed and become eligible for ART, they have often missed days of work due to AIDS-related illness, which worsens if left untreated, leading to increased sick leave or departure from the labor force, and death typically within two years of being diagnosed with AIDS [24], [25], [26], [27].

In contrast, ART rapidly restores physical function and extends life expectancy [28], [29], [30], [31], [32], [33], thereby maintaining worker productivity and keeping families intact [10], [34], [35], [36]. Longitudinal studies among agricultural workers in Kenya [10], [11], [12], [30], [37] and miners in Botswana [35] and Uganda [38] demonstrate a consistent V-shaped pattern for labor force participation and productivity over the course of HIV infection, declining sharply as symptoms worsen in the months before ART initiation and rebounding to near-normal within a few months (Table S2). Studies from India [39], Cambodia [32], Chile [40], Ivory Coast [41], and South Africa [42], [43], [44], [45] also indicate that ART can restore labor productivity. While no studies have followed patients for the length of time modeled in our benefits analysis (10 years), longitudinal studies have not observed any fall off in productivity restored by ART in the first three years after treatment initiation [11].

These findings are tempered by other evidence. For those who lose their jobs upon falling sick with AIDS prior to initiating treatment, lack of employment opportunities in the local labor market can limit the productivity gains due to ART [44]. In addition, some studies that measured output found that patients may not return fully to productivity levels they experienced prior to becoming symptomatic [37].

UNAIDS estimates that worldwide, only seven percent of HIV cases are in persons aged less than 15 years [4]. We therefore assumed that 90 percent of the HIV cases in our cohort were of working age, in order to account for pediatric cases as well as the small fraction of HIV cases that may be occurring in persons who have retired from the labor market due to advancing age. Labor productivity of patients on ART was calculated according to country-specific labor market characteristics, including the fraction of the population that is of working age and the average per capita income [46], [47].

Recent longitudinal studies in southern African countries suggest that middle income groups or the relatively wealthier within a country are at a somewhat higher risk of being infected [48], [49], [50] and more likely to access ART when in need of treatment [49], [51]. Uncertainty in generalizing these findings to all countries in our data set, led us to assume that, in our base case, gross national income (GNI) per working age person is a valid proxy for the productivity of working age HIV-infected persons in the asymptomatic early stage of disease course (i.e. those not in need of treatment). However, using GNI per working age person may overestimate the market value of labor if HIV risk is positively correlated with poverty [52] or if economic output is dominated by natural resource extraction activities that employ a relatively small portion of the labor force [51]. Therefore, we subjected this assumption to sensitivity analysis.

Based on literature review summarized above (and in Information S1), we assumed that the productivity of symptomatic adult HIV-infected persons in need of, but not accessing, ART is reduced to 20 percent of their pre-symptom level. We further assumed that after six months on ART, a patient's productivity is partially restored to 75 percent of pre-symptom level. As shown in Table 1, this level is maintained until one year before death – the period with clinical AIDS that follows ART failure, when productivity again drops to 20 percent pre-symptom level.

Given the uncertainties surrounding these best available estimates of productivity, we conducted sensitivity analyses using a range of values for the assumed productivity of HIV patients with and without ART that were both higher and lower than base case assumptions. The sensitivity analyses separately address: (a) the possibility that HIV-infected persons would, in the absence of HIV infection, tend to engage in less productive work than country average; (b) the extent to which ART restores an HIV-infected person's productivity to the level achieved prior to onset of symptomatic disease; and (c) the extent to which clinical AIDS and pre-AIDS HIV-associated illness resulting from a lack of ART or a failure to respond to ART reduce an infected person's productivity. We separately considered a scenario in which ART patients leaving the workforce due to AIDS are easily replaced from a stock of unemployed workers, and productivity losses due to AIDS are limited to the transition costs (i.e. recruitment and training) of replacing workers (see Information S1).

### Benefits: orphan care costs averted

AIDS deaths have orphaned an estimated 16.6 million children by 2009, of whom 90 percent are in sub-Saharan Africa [1]. Orphans (children with one or more deceased parent) are at higher risk for negative health and educational outcomes [53], generating an urgent need for orphan support including food, clothing, school fees, healthcare, and income generating activities [54], [55]. This support is typically delivered through community programs and/or within households of HIV-infected individuals who foster AIDS orphans [56].

We computed orphan-years averted by per adult life-year gained due to ART using the Spectrum AIDS Impact Model [14] for each of 14 countries, including the 10 with the most Global Fund-supported ART patients and the 10 with the most Global Fund-supported services for orphans and other vulnerable children [18]. Across these 14 countries, between 0.32 and 0.76 orphan-years (average 0.5) are averted each year that an adult patient survives on ART. The number varies across countries because of differences in fertility rates, child mortality and the age distribution of HIV-positive adults. For other Global Fund-supported countries, an estimated number of orphan-years averted per life-year gained on ART was derived from the 14 modeled countries, interpolating linearly based on country-specific fertility rates. For more on the methodology, please see Table 1 and Figure S1.

The averted cost of orphan care was assumed to be $224 per orphan-year, based on a review of service cost data from 300 non-governmental organizations, covering 7400 sites in 22 countries in sub-Saharan Africa [55].

### Benefits: end of life care delayed

We assumed that AIDS patients not accessing ART received end of life care over the last 1.5 years before death from AIDS. The cost of this care averaged $480 per patient not accessing ART, based on $49 worth of non-ARV drugs and the country-specific cost of 9.7 inpatient days and 5.5 outpatient days of clinical care [13], [19], [57]. For patients dying after failure of ART, the additional end-of-life care cost was estimated at one-third of the cost per patient not accessing ART (cross-country average $160), incurred over the last six months of life [7].

All costs and benefits were discounted at three percent per annum as recommended by the WHO [58] and the US Public Health Service [59], and expressed in 2008 US dollars. Undiscounted results are also reported.

## Results

### ART program costs

With (without) discounting, program costs total $14.2 billion ($16.6 billion) [7].

### Benefits of ART

**Figure 2:**
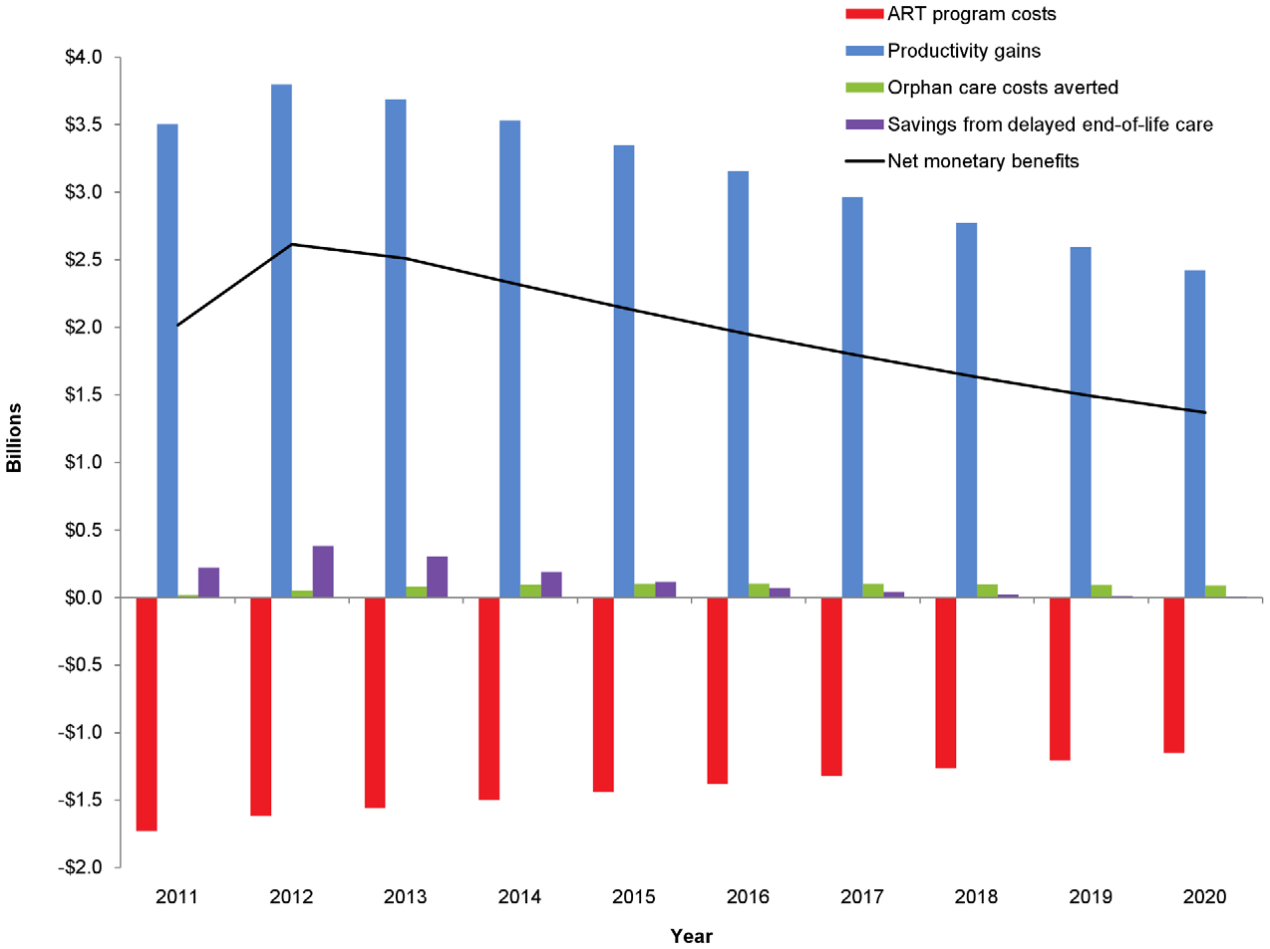
Comparing ART program costs and benefits. Annual discounted ART program costs, productivity gains, orphan care costs averted, and net monetary benefits for the cohort of Global Fund-supported patients on treatment as of 2011.

Over the 10-year period, ART for the 3.5 million patient initial cohort saves a cumulative 18.5 million life-years [7]. With base case assumptions regarding productivity, the corresponding gross discounted economic benefits amount to $34.0 billion (Table 2). Figure 2 illustrates the trends in program costs, productivity gains, and costs averted on orphan and end-of-life care resulting from ART over the 10 years.

**Table 2:** Program costs and economic benefits for the cohort of 3.5 million people on ART in 2011, cumulative 2011–2020 (US$ billions).

Line Item	Base case, discounted	Base case, undiscounted
Program cost	$14.2M	$16.6M
Labor productivity	$31.8M	$37.1M
Orphan care costs averted	$0.83M	$1.0M
End-of-life OI treatment costs averted	$1.4M	$1.5M
Total benefit	$34.0M	$39.6M
Net benefit	$19.8M	$23.0M
Benefit/cost	240%	239%

Productivity gains follow the trend in patients surviving on ART, reaching a maximum in 2012, about a year after the peak in total number of patients on ART in 2011. Across the Global Fund-supported patient cohort, the value of increased labor productivity reaches $3.8 billion per year in 2012, and gradually declines to $2.5 billion per year by 2020 (Figure 2). Over the 10-year study period, the total discounted productivity gain is $32 billion.

The value of averted orphan care increases over time, in direct proportion to the number of life-years added each year for patients receiving ART, as can be seen in Figure 2. The discounted cumulative benefit over the decade is $0.83 billion. The monetary value of delaying end-of-life care is $1.4 billion over the study period, with annual savings peaking in 2012.

### Net benefits of ART

In our base case, the estimated net benefit – the difference between estimated economic benefits and ART program cost – is positive, amounting to $19.8 billion, while the gross benefit ($34.2 billion) equals 240 percent of the program cost over the study period. The benefit-cost ratio was not sensitive to discounting (Table 2).

### Sensitivity Analysis

In Table 3 and 4, we show the results from three-way sensitivity analysis that considers the uncertainty about the magnitude of effect of HIV and ART on productivity. Estimated benefits ranged from 81 percent to 287 percent of the ART program cost. Under most combinations of assumptions about the ability of ART to restore productivity and about the baseline productivity of asymptomatic HIV patients (i.e. the maximal level of productivity that could be restored), the projected costs of ART are fully recouped. Even in the most pessimistic scenario, where the baseline value of productive output of an asymptomatic ART patients is just half of GNI per working age person, and ART only increases an AIDS patient's productivity from 40 percent to 60 percent of baseline productivity, the benefit streams totaled $11.5 billion and offset 81 percent of the cost of ART programs (Table 4).

**Table 3:** Discounted monetary benefits, net benefit (benefits minus program costs), and benefit as a percentage of ART program costs, from restored labor productivity, averted orphan care and delayed end-of-life care combined, cumulative over 2011–2020 (US$ billions) Bold text indicates the base case scenario estimate.

*Productivity of untreated patient in need of ART (as percentage of asymptomatic HIV patient productivity)*		*Productivity of patient responding to treatment (as percentage of asymptomatic HIV patient productivity)*		
		90%	75%	60%
20%	Benefit	$40.7	**$34.0**	$27.3
20%	Net Benefit	$26.5	**$19.8**	$13.1
20%	Benefit/cost	287%	**240%**	192%
40%	Benefit	$38.9	$32.2	$25.5
40%	Net Benefit	$24.8	$18.0	$11.3
40%	Benefit/cost	275%	227%	180%

**Table 4:** Discounted monetary benefits, net benefit (benefits minus program costs), and benefit as a percentage of ART program costs, from restored labor productivity, averted orphan care and end-of-life care combined, cumulative 2011–2020 (US$ billions), with average productivity of asymptomatic HIV infected persons valued at 50% of GNI per person of working age.

*Productivity of untreated patient in need of ART (as percentage of asymptomatic HIV patient productivity)*		*Productivity of patient responding to treatment (as percentage of asymptomatic HIV patient productivity)*		
		90%	75%	60%
20%	Benefit	$20.3	$16.9	$13.6
20%	Net Benefit	$6.1	$2.7	−$0.6
20%	Benefit/cost	143%	119%	96%
40%	Benefit	$18.2	$14.9	$11.5
40%	Net Benefit	$4.1	$0.7	−$2.6
40%	Benefit/cost	129%	105%	81%

Tables S3 and S4 shows sensitivity analyses of ARV costs trends and coverage of viral load monitoring, respectively. If first-line regimen prices decline five percent per year through 2020, and second-line prices fall by 15 percent per year through 2015, program cost reductions amounted to $2.1 billion (15 percent), raising the baseline benefit-cost estimates for ART from 240 percent to 281 percent. The companion paper also explores other possible shifts in program cost, including higher first-line drug costs as a result of the phasing out of stavudine, which would increase program costs by seven percent, and accelerated migration to second-line as a result of increased use of viral load monitoring, raises discounted program costs by $3.5 billion (25 percent). In this case, the benefit-cost estimate falls modestly from 240 percent in the base case to 192 percent.

If patient retention in ART programs were lower than in the base case, both program costs and benefits are reduced and the benefit-cost ratio declines modestly from 240 percent to 226 percent (Table S5). If productivity losses were limited only to the ‘friction costs’ of replacing lost workers from a stock of unemployed persons, the total economic benefits of ART would not fully offset program costs. Still, nearly three-quarters of the program costs would be recouped at the societal level (Table S6).

## Discussion

Our analysis focuses on the valuation of economic benefits and related costs that accrue from maintaining current patients in ART programs being cofinanced by the Global Fund and a range of other complementary domestic and external sources. Though considerable uncertainty remains, and impact at the level of individual countries may vary substantially, we find that the monetary value of productivity gains, orphan care and end-of-life AIDS care costs averted or delayed are likely to exceed ART program costs.

We estimated the economic returns of the ART programs by comparing them to a ‘null scenario’ in which such a treatment effort does not exist [8], [9]. Our results, therefore, correspond to the overall value of ART programs. We did not attempt to directly compute the marginal return to Global Fund's investment in ART, imagining what would have happened without the Fund's financial participation in ART. Nor do we predict what would happen in the coming years if the Global Fund withdrew its support for ART programs – a policy very unlikely to occur. Recent analysis suggests, however, that if donors stopped funding ART programs, only a portion of these resources, perhaps around 40 percent, would be replaced by domestic sources [60].

Our findings provide evidence that large scale ART in low and middle income countries yields a stream of economic benefits that is likely to offset substantially or exceed the costs of delivering AIDS treatment to millions of patients in these countries. With the Global Fund currently supporting on average about a quarter of the program-level costs of ART across the 98 country programs [13], and other donors contributing significant additional portions, these external funders can justifiably argue that their investments are yielding large economic benefits to recipient countries, in addition to the health gains accruing to the millions of patients on AIDS treatment. Similarly, as national governments gradually assume a larger share of the costs of ART programs, they should also see domestic spending on ART as generating large economic benefits that will likely outweigh the costs incurred.

Costs and benefits accrue to a range of stakeholders. Our analysis included the full cost of ART paid for by a combination of donor funds, domestic government revenue, and direct payments of patients or (in rare cases) private insurers. Increased economic productivity directly benefits patient households. The national government also experiences a follow-on benefit from patients returning to work, in the form of increased taxes collected on incomes and other economic activity (e.g. sales taxes), which our analysis did not incorporate. The presented cost savings on orphan care and end-of-life AIDS care are shared between households of patients and their relations and national governments.

In our model, productivity gains from ART increase proportionally with per capita GNI. Although treatment costs are also correlated with GNI, per-patient net benefits are lower for countries with weaker economies or where HIV is concentrated in socioeconomically marginalized subpopulations. While our analysis makes adjustments wherever possible for country-level variation, for many parameters in our model only regional estimates were available. Therefore, we report only aggregate results.

Our findings demonstrate the value of maintaining the current cohort of patients on ART, as we do not estimate economic returns to a further scale up of ART services. Such actions are likely to have favorable benefit-cost profiles, but their exact value will depend on many additional factors that our analysis did not have the capacity to consider. For example, evidence suggests that more productive members of society are more likely to access ART when coverage is low, because of their relatively greater resources, knowledge, and proximity to services [51]. As national ART programs expand, the incremental patients may be relatively less economically productive, and reaching them may be more difficult and expensive. On the other hand, economies of scale in treatment service delivery may offset these other factors that would tend to drive up unit costs and lower benefits.

Our estimates of ART program cost, particularly for service delivery and for orphan care, are limited by shortcomings in the available data. In some cases, it was necessary to adapt findings from studies in a subset of countries to our whole sample. While we used reasonable methods for doing so, only the collection of country-specific data will enable us to refine this analysis of ART's economic impact to the point where precise estimates for individual countries can be generated. Recently established routine tracking of national program expenditures will in the future generate useful data on country variations and time trends in per-patient and program-level costs of ART and other services [61], including effects of ARV drug price declines, changing WHO treatment regimen recommendations [28], and the proportion of patients on first- and second-line ARV regimens. Improved estimation of productivity gains realized in different settings will further benefit from ongoing efforts to enhance monitoring of patient adherence, retention and quality-of-life (including employment) outcomes, especially over the longer term as patients accumulate years on ART.

Future disaggregation of patient retention and productivity effects by gender could also sharpen these estimates of economic returns. As ART coverage is slightly higher in women than in men but labor force participation and wages tend to be lower, gender disaggregation could be expected to lower the estimated benefits somewhat. However, the impact of ART on non-monetized activities disproportionately performed by women in the household and informal sector – which are not captured in our analysis – is likely to be substantial.

Our assessment of the benefits of ART presented here should be taken as a first approximation of the magnitude of the economic returns to investments in AIDS treatment. While productivity gains, orphan benefits, and cost offsets within clinical HIV care are the most tangible returns on investment that policy-makers may consider when evaluating the affordability of ART programs in the future, these benefit streams capture only a fraction of wider economic, social and health benefits from AIDS therapy. Accounting for second-order negative economic effects of AIDS that may be mitigated by ART, such as a slowing of economic growth due to reduced savings and investment, erosion of human capital, and lower expected lifetime earnings of children who must miss school to care for, or replace the earnings of, a sick parent, would increase the economic benefits we have estimated here.

Economists have attempted to capture these second-order effects and measure the impact of AIDS on economic growth using macroeconomic approaches that simulate the entire economy of a country in computable general equilibrium models. These studies suggest that, in the absence of effective treatment, substantial productivity losses – on the order of a one percent reduction in gross domestic product (GDP) growth per year – could occur in countries with generalized HIV epidemics [6], [49], [62], [63], [64], [65], [66].

Our analysis, like these macroeconomic modeling approaches, does not attempt to measure the full social welfare impact of ART. Such an exercise would likely show that benefits derived from ART are greater than the productivity gains and cost offsets in orphan and end-of-life care [67]. The WHO's benchmarks for cost-effectiveness of averting disability-adjusted life years imply that an incremental life year gained has a monetary value equivalent to a multiple (one to three times) of GDP per capita [68]. Likewise, studies of the ‘value of statistical life’, generally find that people value life years gained at an amount that is greater than their expected income in those years of life, although private willingness-to-pay for mortality risk reductions varies with country, income and age [69], [70].

Despite the restriction of our analysis to a set of first-order economic benefits, the findings presented in this paper underscore the value to low and middle income countries and their external partners of continuing funding for AIDS treatment programs, beyond the moral and social arguments that many have advanced. Progress in delivering high-quality treatment services more efficiently over the next few years, thereby lowering the average cost per patient-year of ART, will help to further raise the benefit-cost balance and thus the economic rationale for investing in this area.

## Supporting Information

Figure S1 **Correlation between Total Fertility Rate and years of ART required to avert one orphan-year.** The number of patient-years of ART required to avert one year of orphanhood was modeled for 14 key countries with ART programs supported by the Global Fund, using the Spectrum model [1]. Together, these countries are responsible for 69 percent of Global Fund ART patients and 94 percent of Global Fund-supported OVC services: Ethiopia, Malawi, Tanzania, Rwanda, Cambodia, India, Burundi, Kenya, Lesotho, Nigeria, South Africa, Uganda, and Zambia. To estimate years of ART required to avert one orphan-year for all other Global Fund-supported countries, we extrapolated the findings from the 14 modeled countries by linear interpolation based on country-specific fertility rate.
(TIF)

Table S1 **Countries with Global Fund supported ART programs.**
(DOCX)

Table S2 **Longitudinal studies of labor force participation and productivity of adult HIV/AIDS patients, with and without ART.**
(DOCX)

Table S3 **Sensitivity analysis of program cost assumptions.**
(DOCX)

Table S4 **Sensitivity of program cost estimates to trends in ARV price and coverage of viral load monitoring.**
(DOCX)

Table S5 **Sensitivity analysis of patient retention on ART.**
(DOCX)

Table S6 **Program costs and economic benefits for the cohort of 3.5 million people on ART in 2011, Results of sensitivity analyses, cumulative 2011–2020 (US$ billions).**
(DOCX)

Information S1 **An elaboration on Materials and Methods.**
(DOCX)
