# Supplementary figures and images for: Economic Returns to Investment in AIDS Treatment in Low and Middle Income Countries
Source: PLoS One. 2011 Oct 5;6(10):e25310. doi: 10.1371/journal.pone.0025310 (PMC3187775; doi:10.1371/journal.pone.0025310)

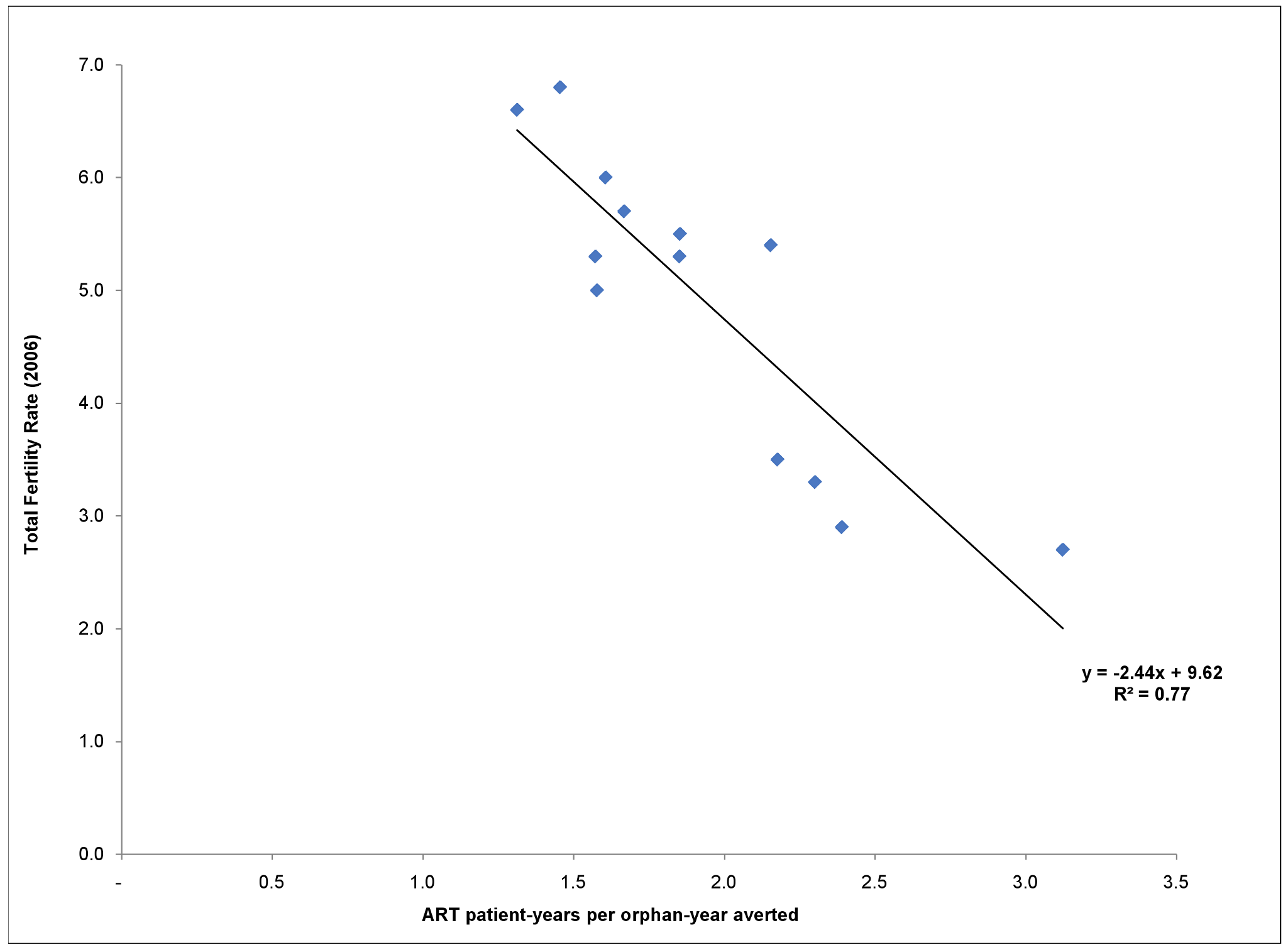

Supplement: Figure S1 — Correlation between Total Fertility Rate and years of ART required to avert one orphan-year. The number of patient-years of ART required to avert one year of orphanhood was modeled for 14 key countries with ART programs supported by the Global Fund, using the Spectrum model [1]. Together, these countries are responsible for 69 percent of Global Fund ART patients and 94 percent of Global Fund-supported OVC services: Ethiopia, Malawi, Tanzania, Rwanda, Cambodia, India, Burundi, Kenya, Lesotho, Nigeria, South Africa, Uganda, and Zambia. To estimate years of ART required to avert one orphan-year for all other Global Fund-supported countries, we extrapolated the findings from the 14 modeled countries by linear interpolation based on country-specific fertility rate. (TIF) [file pone.0025310.s001.tif]
