# Supplementary material for: Economic Returns to Investment in AIDS Treatment in Low and Middle Income Countries
Source: PLoS One. 2011 Oct 5;6(10):e25310. doi: 10.1371/journal.pone.0025310 (PMC3187775; doi:10.1371/journal.pone.0025310)
Supplement: Table S1 — Countries with Global Fund supported ART programs. (DOCX) [file pone.0025310.s002.docx]

**Table S1. Countries with Global Fund supported ART programs.** Listed in order of program size. Patient numbers only reflect those patients that countries report are in programs receiving Global Fund support. For some countries, this amount is less than the total number of patients enrolled in ART.

| Country | Region | ART Patients 2011 [35,36] | GNI per capita, Atlas Method 2008 [32] | Percent of Population that is Working age [32] |
| --- | --- | --- | --- | --- |
| Nigeria | AFR | 418,150 | $1,160 | 54% |
| India | SEAR | 352,509 | $1,070 | 63% |
| Zambia | AFR | 320,843 | $950 | 51% |
| Tanzania | AFR | 283,965 | $440 | 52% |
| Malawi | AFR | 237,936 | $290 | 50% |
| Ethiopia | AFR | 228,334 | $280 | 53% |
| Mozambique | AFR | 225,919 | $370 | 53% |
| Kenya | AFR | 127,904 | $770 | 55% |
| Rwanda | AFR | 103,270 | $410 | 55% |
| Uganda | AFR | 101,235 | $420 | 48% |
| Namibia | AFR | 98,801 | $4,200 | 59% |
| Russian Federation | EUR | 92,814 | $9,620 | 72% |
| Cameroon | AFR | 84,265 | $1,150 | 55% |
| China | WPR | 84,058 | $2,940 | 72% |
| Zimbabwe | AFR | 67,737 | $1,355 | 56% |
| Lesotho | AFR | 64,631 | $1,080 | 56% |
| Swaziland | AFR | 58,263 | $2,520 | 57% |
| Cambodia | WPR | 46,599 | $600 | 62% |
| Congo, Dem. Rep. | AFR | 44,237 | $1,970 | 56% |
| Ghana | AFR | 39,739 | $670 | 58% |
| Burkina Faso | AFR | 29,519 | $480 | 52% |
| Mali | AFR | 23,936 | $580 | 53% |
| Burundi | AFR | 22,078 | $140 | 58% |
| Haiti | CA/Car | 18,500 | $660 | 59% |
| Angola | AFR | 18,199 | $3,450 | 52% |
| Dominican Republic | CA/Car | 17,898 | $4,390 | 62% |
| Central African Republic | AFR | 17,395 | $410 | 55% |
| Indonesia | SEAR | 17,223 | $2,010 | 67% |
| Benin | AFR | 16,908 | $690 | 54% |
| Cote d'Ivoire | AFR | 15,750 | $980 | 55% |
| South Africa | AFR | 15,006 | $5,820 | 65% |
| Peru | LA | 14,784 | $3,990 | 64% |
| Congo, Rep. | AFR | 12,445 | $150 | 50% |
| Vietnam | WPR | 11,532 | $890 | 67% |
| Honduras | CA/Car | 9,036 | $1,800 | 58% |
| Thailand | SEAR | 8,681 | $2,840 | 71% |
| Guinea | AFR | 8,247 | $1,355 | 54% |
| Guatemala | CA/Car | 8,150 | $2,680 | 68% |
| Jamaica | CA/Car | 7,915 | $4,870 | 62% |
| El Salvador | CA/Car | 7,308 | $3,480 | 60% |
| Niger | AFR | 6,951 | $330 | 48% |
| Papua New Guinea | WPR | 6,770 | $1,010 | 57% |
| Eritrea | AFR | 6,197 | $300 | 56% |
| Senegal | AFR | 5,852 | $970 | 54% |
| Ecuador | CA/Car | 5,705 | $3,640 | 62% |
| Sudan | EMR | 5,528 | $1,130 | 57% |
| Sierra Leone | AFR | 5,409 | $320 | 55% |
| Gabon | AFR | 5,125 | $7,240 | 59% |
| Chad | AFR | 5,051 | $530 | 51% |
| Cuba | CA/Car | 5,000 | $1,355 | 70% |
| Nepal | SEAR | 4,516 | $400 | 59% |
| Guyana | CA/Car | 3,832 | $1,420 | 64% |
| Liberia | AFR | 3,501 | $170 | 54% |
| Morocco | EMR | 3,458 | $2,580 | 66% |
| Guinea-Bissau | AFR | 3,147 | $250 | 54% |
| Uzbekistan | EUR | 3,039 | $910 | 65% |
| Togo | AFR | 2,867 | $400 | 56% |
| Paraguay | LA | 2,600 | $2,180 | 61% |
| Equatorial Guinea | AFR | 2,143 | $14,980 | 56% |
| Belarus | EUR | 2,083 | $5,380 | 71% |
| Ukraine | EUR | 1,615 | $3,210 | 70% |
| Lao PDR | WPR | 1,603 | $740 | 58% |
| Moldova | EUR | 1,466 | $1,470 | 72% |
| Pakistan | EMR | 1,463 | $980 | 59% |
| Mauritania | AFR | 1,439 | $1,355 | 58% |
| Nicaragua | CA/Car | 1,362 | $1,080 | 60% |
| Suriname | CA/Car | 1,313 | $4,990 | 65% |
| Bolivia | CA/Car | 1,196 | $1,460 | 59% |
| Djibouti | EMR | 1,142 | $1,130 | 60% |
| Gambia, The | AFR | 1,123 | $390 | 55% |
| Kazakhstan | EUR | 1,044 | $6,140 | 61% |
| Belize | CA/Car | 913 | $3,820 | 60% |
| Philippines | WPR | 844 | $1,890 | 62% |
| Georgia | EUR | 819 | $2,470 | 68% |
| Somalia | EMR | 809 | $1,327 | 52% |
| Tunisia | EMR | 455 | $3,290 | 70% |
| Egypt, Arab Rep. | EMR | 438 | $1,800 | 63% |
| Tajikistan | EUR | 416 | $600 | 59% |
| Bulgaria | EUR | 381 | $5,490 | 69% |
| Azerbaijan | EUR | 357 | $3,830 | 69% |
| Bangladesh | SEAR | 270 | $520 | 64% |
| Yemen, Rep. | EMR | 265 | $950 | 53% |
| Kyrgyz Republic | EUR | 260 | $740 | 65% |
| Sri Lanka | SEAR | 234 | $1,780 | 68% |
| Madagascar | AFR | 202 | $410 | 54% |
| Armenia | EUR | 140 | $3,350 | 68% |
| Iran, Islamic Rep. | EMR | 136 | $3,297 | 71% |
| Albania | EUR | 118 | $3,840 | 66% |
| Jordan | EMR | 80 | $3,310 | 63% |
| Bosnia and Herzegovina | EUR | 46 | $4,510 | 71% |
| Timor-Leste | SEAR | 42 | $2,460 | 52% |
| Bhutan | SEAR | 41 | $1,900 | 64% |
| Montenegro | EUR | 35 | $6,440 | 68% |
| Macedonia, FYR | EUR | 32 | $4,140 | 70% |
| Sao Tome and Principe | AFR | 14 | $1,020 | 55% |
| Comoros | AFR | 13 | $750 | 59% |
| Mongolia | WPR | 13 | $1,680 | 70% |
| Maldives | SEAR | 3 | $3,630 | 67% |
