# Supplementary material for: Economic Returns to Investment in AIDS Treatment in Low and Middle Income Countries
Source: PLoS One. 2011 Oct 5;6(10):e25310. doi: 10.1371/journal.pone.0025310 (PMC3187775; doi:10.1371/journal.pone.0025310)
Supplement: Table S2 — Longitudinal studies of labor force participation and productivity of adult HIV/AIDS patients, with and without ART. (DOCX) [file pone.0025310.s003.docx]

**Table S2. Studies of labor force participation and productivity of adult HIV/AIDS patients, with and without ART**

| **Source** | **Country, time** | **Population initiating ART** | **Follow-up period** | **Design** | **Comparison group** | **Employment / labor force participation** | **Hours worked, absenteeism, output per work hour** |
| --- | --- | --- | --- | --- | --- | --- | --- |
| [37] | Rural KENYA, 2004-2005 | 266 patients started ART in past 100 days | 6 months | 2 household surveys | 503 non-ART household members | Pre-ART 65%; post-ART 82% (formal and informal sectors) | Pre-ART 20.3 hours; post-ART 28.2 hours per week |
| [26] | INDIA, Kerala, 2004-2005 | 136 patients | 10 months | Longitudinal; pre-post ART initiation, patient surveys | N/A | Pre-ART 27%; post-ART 74% |  |
| [38] | Township, SOUTH AFRICA, 2002 | 137 patients, of whom 104 retained | 1 year | Socio-economic surveys | 1,536 community members | Pre-ART labor force participation 66%, employment 42%; post-ART labor force participation 85%, employment 53% |  |
| [27] | CAMBODIA, 2005-2008 | 549 patients (4% AIDS-related deaths) | 1 year | Surveys of health-related quality of life | N/A | Pre-ART 49%; post-ART 96% |  |
| [22] | INDIA, Tamil Nadu, 2005-2007 | 452 patients | 1.5 years | Socio-economic surveys, 6 month intervals | 867 patients receiving non-ART care | Post-ART: Increase by 26% at 6 months, sustained at 1 year | Post-ART: Increase from 12.7 hours per week at baseline to 25.5 hours per week, at 6 months and sustained at 1 year |
| [20] | BOTSWANA, 2001-2006 record review | 441 diamond mine workers (82% male) | 4 years | Retrospective pre-post; company record review | 89 patients not initiating ART |  | Absence pre-ART peaks at 5 days missed in the month prior to ART initiation; within 6 months on ART absenteeism dropped to level observed among workers not enrolled in ART. Effect sustained up to 4 years on ART. |
| [21,25] | KENYA tea plantation, 2004-2006 | 97 workers (42% male) | 1 year | Company record review | 2485 matched workers of unknown HIV status (68% men) |  | 9 months & 1 week pre-ART 15% and 65% fewer days worked, respectively. At 7 months post-ART, men regained productivity of matched controls; women remained 10-15% less productive. |
| [21] | KENYA tea plantation, 1997-2002 | N/A | N/A | Company record review | 54 HIV-infected & 271 uninfected workers (61% male) | Untreated patients earned 18% less in year preceding AIDS-related end of employment | Untreated symptomatic patients worked <50% as much as controls |
| [29] | IVORY COAST, 1999-2001 | 74 electric company workers | 2 years | Company & medical record review | 3,394 workers |  | Pre-ART: Sick leave 0.16 months/year (0.14 in controls). Pre-ART 12% ‘fully functioning’; post-ART 84% at 12 months and 91% at 24 months |
| [30] | SOUTH AFRICA, (dates not reported) | 2,400 employees | 1.5 years | Monitoring by employer-sponsored ART program | N/A |  | Absence pre-ART 7.5 days/month; post-ART 2.9 days at 6 months, 2.1 days at 18 months |
| [28] | CHILE, 8 public hospitals, 2000-2001 | 512 patients (80% men) | 2.3 years | Longitudinal; pre-post ART initiation, patient surveys | N/A |  | Absence pre-ART 78 days/year; post-ART 52 days/year at 28 months |
| [31] | SOUTH AFRICA, 3 urban clinics | 447 patients (21% male) | 0.5 year | Patient surveys | 452 pre-ART patients (21% male) |  | Absence pre-ART 3.1 days/month; post-ART 1.3/month at 3-6 months |
| [39] | UGANDA, 1 urban and 1 rural clinic | 602 patients (32% male) | N/A | Multivariate regression analysis of baseline data of a prospective cohort, patient surveys | CD4 250-400 compared to CD4 <250 at baseline | Job loss and worked in last 7 days strongly associated with CD4. WHO Stage 3+ (47% worked), WHO Stage 2- (67% worked) |  |

Note: In patients initiating ART, when reported, the population median CD4 count at ART initiation was between 145 cells/uL (Kenya [21,24]) and 165 cells/uL (Botswana [20]). ‘Patients’ denotes HIV-infected patients; post-ART = from time of ART initiation; N/A = not applicable.
