# Supplementary material for: Economic Returns to Investment in AIDS Treatment in Low and Middle Income Countries
Source: PLoS One. 2011 Oct 5;6(10):e25310. doi: 10.1371/journal.pone.0025310 (PMC3187775; doi:10.1371/journal.pone.0025310)
Supplement: Table S3 — Sensitivity analysis of program cost assumptions. (DOCX) [file pone.0025310.s004.docx]

**Table S3. Sensitivity analysis of program cost assumptions**. No changes in health impact were assumed.

|  | Base-case | First-line ARV cost decline by 5% per year through 2020 and second-line ARV cost declines 15% per year until 2015 | Viral load monitoring uptake increases transition rate to second-line regimens from 2.6% per year to 6% per year |
| --- | --- | --- | --- |
| Program Cost | $14.2B | $12.1B | $17.7B |
| Labor productivity | $31.8B | Same (as base-case) | Same (as base-case) |
| Orphan care costs averted | $0.83B | Same (as base-case) | Same (as base-case) |
| End-of-life OI treatment costs averted | $1.4B | Same (as base-case) | Same (as base-case) |
| Total benefit | $34.0B | Same (as base-case) | Same (as base-case) |
| Net benefit | $19.8B | $21.9B | $16.3B |
| Benefit/cost | 240% | 281% | 192% |
