# Supplementary material for: Economic Returns to Investment in AIDS Treatment in Low and Middle Income Countries
Source: PLoS One. 2011 Oct 5;6(10):e25310. doi: 10.1371/journal.pone.0025310 (PMC3187775; doi:10.1371/journal.pone.0025310)
Supplement: Table S4 — Sensitivity of program cost estimates to trends in ARV price and coverage of viral load monitoring. (DOCX) [file pone.0025310.s005.docx]

**Table S4. Sensitivity of program cost estimates to trends in ARV price and coverage of viral load monitoring**

| **Alternative assumptions** | **Change in 10-year program cost (discounted)** | **Change as a percent of base case** |
| --- | --- | --- |
| First-line ARV cost decline by 5% per year through 2020 and second-line ARV cost declines 15% per year until 2015 | $2.1 billion | 15% |
| Viral load monitoring uptake increases transition rate to second-line regimens from 2.6% per year to 6% per year | $3.5 billion | 25% |
