# Supplementary material for: Economic Returns to Investment in AIDS Treatment in Low and Middle Income Countries
Source: PLoS One. 2011 Oct 5;6(10):e25310. doi: 10.1371/journal.pone.0025310 (PMC3187775; doi:10.1371/journal.pone.0025310)
Supplement: Table S5 — Sensitivity analysis of patient retention on ART. (DOCX) [file pone.0025310.s006.docx]

**Table S5. Sensitivity analysis of patient retention on ART.**

| **Indicator** | **Base case** | **Alternative** | **Difference** | **Difference as a percent of base case** |
| --- | --- | --- | --- | --- |
| Annual probability of remaining on ART after 1 year | 96% | 90% | 6% |  |
| Program Cost | $14.2 B | $ 11.2B | $ 3.0B | 21% |
| Gross Benefits | $34.0B | $ 25.3B | $ 8.7B | 26% |
| Net Benefit | $19.8B | $ 14.1B | $ 5.7B | 29% |
| Benefit/Cost Ratio | 240% | 226% |  |  |
