# Supplementary material for: Economic Returns to Investment in AIDS Treatment in Low and Middle Income Countries
Source: PLoS One. 2011 Oct 5;6(10):e25310. doi: 10.1371/journal.pone.0025310 (PMC3187775; doi:10.1371/journal.pone.0025310)
Supplement: Table S6 — Program costs and economic benefits for the cohort of 3.5 million people on ART in 2011, Results of sensitivity analyses, cumulative 2011–2020 (US$ billions). (DOCX) [file pone.0025310.s007.docx]

**Table S6. Program costs and economic benefits for the cohort of 3.5 million people on ART in 2011, Results of sensitivity analyses, cumulative 2011-2020 (US$ billions)**

| **Line Item** | **Base case (human capital approach), discounted** | **Friction cost approximation, discounted** |
| --- | --- | --- |
| Program cost | $14.2B | $14.2B |
| Labor productivity | $31.8B | $8.03B |
| Orphan care costs averted | $0.83B | $0.83B |
| End-of-life OI treatment costs averted | $1.4B | $1.4B |
| Total benefit | $34.0B | $10.2B |
| Net benefit | $19.8B | $(3.9B) |
| Benefit/cost | 240% | 72% |
