# Supplementary material for: Economic Returns to Investment in AIDS Treatment in Low and Middle Income Countries
Source: PLoS One. 2011 Oct 5;6(10):e25310. doi: 10.1371/journal.pone.0025310 (PMC3187775; doi:10.1371/journal.pone.0025310)
Supplement: Information S1 — An elaboration on Materials and Methods. (DOCX) [file pone.0025310.s008.docx]

**Supporting Information**

## Economic Returns to Investment in AIDS Treatment in Low and Middle Income Countries

**Authors and Affiliations**

Corresponding Author: Stephen Resch^1^

Authors: Stephen Resch^1,^ Eline Korenromp^2,3^, John Stover^4^, Matthew Blakley^2^, Carleigh Krubiner ^5^, Robert Hecht^5^, Rifat Atun^2,6^

Affiliations:

^1^Harvard School of Public Health, Center for Health Decision Science, 718 Huntington Avenue, Boston, Massachusetts 02130, USA, stephen_resch@harvard.edu

^2^The Global Fund to Fight AIDS, Tuberculosis and Malaria, Geneva, Switzerland

^3^Department of Public Health, University Medical Center, Rotterdam, the Netherlands

^4^Futures Institute, Glastonbury, CT, USA

^5^Results for Development Institute, Washington, DC, USA

^6^Imperial College London, UK

**An Elaboration on Materials and Methods**

**Treatment Cohort Size: Survival with and without ART**

The treatment cohort contains patients in national ART programs in 98 countries that are co-financed by Global Fund (Table S1).

Treatment cohort survival of adult HIV/AIDS patients with and without ART was modeled using established HIV/AIDS epidemiological methods and assumptions [1,2]. For a cohort of HIV-infected patients starting ART in a given year, the number of patients surviving in each future year is determined from the number of patients in the previous year and the survival to the following year. Survival in the first year is assumed on treatment of 79.5 percent and in subsequent years of 95.8 percent, for all countries based on retention rates reported to WHO by national AIDS programs in high-burden countries in 2008 [3,4]. Survival estimates incorporated an assumption that patients would initiate and receive treatment according to WHO-recommended treatment eligibility criteria [5]. Average survival on ART was 12 years.

Patients with disease sufficiently progressed to meet treatment eligibility criteria face poor survival rates if they cannot access ART. The model we used leveraged data regarding the natural history of disease progression and survival of untreated patients to estimate a survival function for patients (hypothetically) withdrawn from treatment [6,7,8]. We modeled this (hypothetical) counterfactual situation in order to compute incremental life-years attributable to ART and corresponding productivity and orphanhood outcomes. The number of people that would still be alive over subsequent calendar years was calculated and compared to numbers still alive and on ART, to estimate numbers of deaths averted and life-years saved in each calendar year, as can be seen in Figure 1 (in the main text).

In the counterfactual scenario that these patients in need would not continue to receive ART, we calculated mortality based on survival data for people meeting criteria for ART, but who were never on treatment. Based on the time from infection to AIDS deaths as analyzed by the ALPHA network, a collaboration of cohort studies in Africa [1,2], the cumulative proportion of a cohort currently on ART that would die if ART were stopped would be 18 percent after one year, 46 percent after two years, 64 percent after three years, 76 percent after four years, 84 percent after five years, and 97 percent after 6 years – with median survival being three years. Using these cumulative mortality rates in a Weibull survival curve [1], we calculated the number of people that would still be alive over subsequent calendar years with and without ART, to estimate numbers of deaths averted and life-years saved in each calendar year. Although most patients on treatment in 2011 started treatment in earlier years and thus may have improved immune systems, we assumed that if treatment were stopped abruptly they would have survival patterns similar to those who became eligible for treatment but never received it, so survive for a median slightly more than two years after the cessation of treatment [2].

**Orphan-years averted**

Orphan-years averted by ART were computed per adult life-year gained on ART, using the *Spectrum AIDS Impact Model* [9]. For each of the 14 countries that include the 10 countries with the most Global Fund-supported patients on ART and the 10 countries with the most Global Fund-supported services for orphans and vulnerable children (OVC), the model was run to determine numbers of orphans averted by the ART program. These countries represent 69 percent of ART patients and 94 percent of all Global Fund-supported OVC services. Orphans were defined as children losing one or both parents. The number of children orphaned each year was used to compute corresponding number of orphan-years, counting each orphan for the years remaining until the age of 18, accounting for age-specific mortality.

The difference in the number of orphan-years with and without ART was divided by the patient-years of ART to estimate an average number of orphan-years averted per patient-year of ART. Across the 14 modeled countries, between 0.32 and 0.76 orphan-years (average 0.5) are averted each year that a patient survives on ART. The number varies across countries because of country variations in fertility rates, infant mortality and the age distribution of HIV-positive adults. For other Global Fund-supported countries, we extrapolated the findings from the 14 modeled countries by linear interpolation based on country-specific fertility rate. The relationship between national average total fertility rate and number of patient-years on ART required to avert one orphan-year is shown in Figure S1.

**Estimation of Treatment Program Cost**

For ART, a year of first-line treatment for an adult is estimated to cost $487, of which ARV drugs make up the largest cost component ($204). Second-line ART was estimated to cost an average of $1,521 per adult patient-year (of which ARV drugs make up $1,238). These costs represent overall recurrent program-level service delivery costs, to which the Global Fund contributes alongside other partners and domestic resources. Drug costs are based on procurement prices reported by Global Fund-supported countries, whereas the costs of treatment delivery were estimated based on data from comprehensive costing studies available from selected countries [2]. Proportions of patients on first-line and second-line regimens were estimated using treatment use data reported by national AIDS programs to WHO in 2008 [3,4]. We conducted sensitivity analysis on the cost of ARVs and treatment service delivery. Specifically we considered a scenario in which first-line regimen prices decline 5 percent per year through 2020, and second-line prices fall by 15 percent per year through 2015 (Table S3). In a separate analysis, we considered the impact of increased rates of transition to second-line therapies as a result of viral load testing to detect virologic failure (Table S4). Table S5 shows the results of a sensitivity analysis in which the retention in treatment is lower than in the base case. The benefit-cost ratio is reduced only modestly because the patients dropping out experience less benefit, but also lower program costs.

**Measuring and Valuing Productivity Gains from ART**

*Human capital versus friction approach*

Like most studies of the impact of disease on productivity, we used the *human capital approach* to valuing productivity, in which the value of work is the expected earnings from work [10]. In this method, when a worker stops working because of illness or illness-related premature death, there is a productivity loss equal to the expected earnings for that worker in the event that they had not gotten ill.

Untreated HIV/AIDS patients with symptomatic disease (i.e. that meet eligibility criteria for treatment) are more likely to leave the workforce, reduce their hours at work, and reduce their output per hour worked. It is clear that these reductions are a productivity loss from the perspective of individual patients and their families, but in certain cases the impact on society may be less severe. When unemployment levels are very high, there is a ready pool of workers available to replace those who fall sick and die. While the family of the replaced worker may be worse off, the family of the replacement worker will be better off, so at the societal level the productivity loss may be less than measured by the human capital approach. Indeed, some macroeconomic models developed in the pre-ART era have predicted that the AIDS-related mortality may have the effect of increasing *per capita* income of some countries because nearly the same amount of work is being done, but, because of AIDS deaths, there are fewer people (i.e. the numerator for per capita income is unchanged, but the denominator is smaller) [11].

The *friction cost approach* [12] has been proposed and applied in several studies as an alternative method to the human capital approach to measuring productivity. The distinguishing feature of this approach is the assumption that in settings with sufficient pool of unemployed workers, someone currently unemployed will replace a worker who is stricken with disease. There is a ’friction period’, spanning the transition from one worker to another, during which there is lost productivity and costs related to recruiting and training new workers, but these ‘friction costs’ are usually much lower than the lost productivity of the sick worker that would have been estimated with the human capital approach. The friction cost method has some intuitive appeal for settings with large pools of unemployed unskilled wage workers. But the method has generated criticism for theoretical shortcomings (e.g. [13]). Moreover, the friction cost approach is not applicable in the case of full employment economies or subsistence farming where there is no replacement for the workers lost [14].

We explored the possibility of applying the friction cost approach to the unskilled wage labor portion of economies in countries with high levels of unemployment, but did not pursue it in depth due to limitations in data regarding the distribution of HIV-infected workers across sectors of the economy, skill level of HIV-infected workers, and country-and sector-level recruitment and training costs. However, to provide some insight into how this alternative approach might impact the results, we considered the case in which a portion of workers is replaced by those who are unemployed.

*Productivity in treated and untreated HIV/AIDS patients*

We estimated productivity loss in AIDS patients and productivity among those receiving ART based on a review of published studies described in Table S2. By the time HIV-infected workers are diagnosed and become eligible for ART, they have often missed days of work due to AIDS-related illness, which worsens dramatically if left untreated, leading to increased sick leave or departure from the labor force, as well as death, typically within two years of being diagnosed with AIDS [15,16,17]. In contrast, ART restores physical function and extends life expectancy [18,19], thereby maintaining worker productivity and keeping families intact [20,21,22,23]. Longitudinal studies among agricultural workers in Kenya [23,24,25] and miners in Botswana [20] show a consistent V-shaped pattern for labor force participation and productivity over the course of HIV infection, declining sharply as symptoms worsen in the months before ART initiation and rebounding to near-normal within a few months after ART initiation. Studies from India [26], Cambodia [27], Chile[28], Ivory Coast [29], and South Africa[30,31] also consistently show that ART can restore or nearly restore labor productivity within months, and sustain this effect through at least the first one to three years after ART initiation. While no studies have followed patients for the length of time modeled in our benefits analysis (11 years), longitudinal studies have not observed any fall-off in productivity restored by ART within the first three years after treatment initiation [25].

*Calculation of productivity gains at country-level: human capital approach*

For each country, the productivity gain due to ART (**PG_ART_** ) in each year **y** of the time horizon was computed by estimating the productivity of the ART patients and subtracting the productivity that would have been realized in that year had there been no treatment available. Productivity is the product of additional work time (i.e. incremental full-time equivalents of labor) and country-specific gross national income per working age person. In using GNI per working age person, our estimates account for labor force participation and unemployment. These parameters were obtained from the publicly available World Development Indicators compiled by the World Bank [32]. Data from the most recent year available was used. In most cases, this was year 2008. When a value was missing for a specific country, we imputed the value by estimating the population-weighted average among all countries in the same WHO region for which data were available.

Additional work time was calculated by dividing the patient population into a high-productivity group consisting of those patients responding to ART and a low-productivity group consisting of untreated patients and ART patients in their last year of life (and therefore assumed to have failed treatment) and those in the first 6 months following ART initiation (who have not yet experienced a restoration of functional status). The productivity *levels* of these two groups relative to country average, L_hi_ and L_lo_, respectively, were assumed based on a review, outlined in Table S1, of studies regarding the productivity of treated and untreated HIV patients. In our base case, L_hi_ was 0.75 and L_lo_ was 0.2.

**Productivity Gain Due to ART (PG_ART_):**

**PG_ART_** = **a** × **b** × (**PP_ART_** - **PP_NoART_**) × **GNI per capita** / **%WA**

*where*

**PP_ART_** is the potential productivity of patients on ART

PP_ART_ = ( [Patients Alive in Year y]_ART_ – [Patients Dying in Year y+1]_ART_ – (c × [Patients Initiating ART in Year y]_ART_)) ×[L_hi_] + ( [Patients Dying in Year y+1]_ART_ + (c × [Patients Initiating ART in Year y]_ART_)) ×[L_lo_]

**PP_NoART_** is the (counterfactual) potential productivity of patients without ART

PP_NoART_ = ( [Patients Alive in Year y]_NoART_ ) × [L_lo_]

**a** is the relative productivity of the HIV-infected population, prior to experiencing HIV-related symptoms. In the base case, this is assumed to be 1. In the sensitivity analysis, we present results for the case where a = 0.5.

**b** is the fraction of ART patients that are of working age. This is assumed to be 0.9.

**c** is the number of years after ART initiation before productivity is restored. This is assumed to be 0.5 years.

**L_hi_** is the potential productivity level (relative to country-average) for ART patients responding to treatment; between 0 and 1. In the base case, this is assumed to be 0.75. In the sensitivity analysis, 0.6 and 0.9 are used as upper and lower bounds.

**L_lo_** is the potential productivity level (relative to country-average) for untreated patients who are treatment-eligible and ART patients in whom treatment has failed; between 0 and 1. In the base case, this is assumed to be 0.2. In the sensitivity analysis, 0.4 is used as an upper bound.

**%WA** is the country-specific fraction of the population that is of working age, derived from the dependency ratio [32].

**GNI** **per** **capita** is country-specific per capita gross national income (Atlas method) in 2008 US dollars divided by total population [32].

*Calculation of productivity gains at country-level: friction cost approximation approach*

For this sensitivity analysis we assumed that two-thirds of workers who die from AIDS can be replaced for a cost equal to six months wages. For the other one-third of workers, we assumed that due to the scarcity of their skills, the loss was equal to five years. On average, the productivity losses were therefore two years per person dying from AIDS. For simplicity, the losses were assumed to accrue to the year in which the person died. Results are shown in Table S6. Productivity gains are reduced from the base case by 75 percent. As a result, the net benefits are negative. However, the total benefits from all three sources still offset over 70 percent of the costs of the ART program.

**Cost of Orphan Care**

The social and economic effects on children orphaned by AIDS deaths of parents include poorer nutrition, poorer education, increased vulnerability to child labor and disease, and lower future earnings once grown-up [33,34]. Instead of quantifying all these effects throughout the lifespan of orphans, we focused on the direct costs of providing orphan care and support through social mitigation programs. Orphan support was estimated to cost a mean $224 per child-year, based on cost data collected from 300 orphan care service providers at 7400 sites in 22 African countries [9]. This estimate included a downward adjustment from observed costs for economies of scale expected during ongoing program scale-up. As in [9], we assumed that only orphans in families below the country’s poverty line would require support services. In wealthier families, the care of orphans would likely be absorbed without assistance of social programs.

These orphan care costs would be saved by donors and governments if ART programs prevent children from becoming orphans. Where orphan care programs have not been scaled-up, the impact of ART may not directly translate into savings in the budgets of orphan care programs. Nevertheless, the benefits we calculate can be considered to represent a lower bound of the economic value of reducing orphanhood, because the economic cost of *un-*mitigated negative outcomes for orphans (i.e. negative impacts not reversed by social programs) is probably larger than the cost of mitigation programs.

**References**

1. Stover J, Johnson P, Zaba B, Zwahlen M, Dabis F, et al. (2008) The Spectrum projection package: improvements in estimating mortality, ART needs, PMTCT impact and uncertainty bounds. Sex Transm Infect 84 Suppl 1: i24-i30.

2. Stover J, Korenromp EL, Blakley M, Komatsu R, Viisainen K, et al. (2011) Long-term costs and health impact of continued global fund support for antiretroviral therapy. PLoS One 6: e21048.

3. Renaud-Thery F, Avila-Figueroa C, Stover J, Thierry S, Vitoria M, et al. (2011) Utilization patterns and projected demand of antiretroviral drugs in low- and middle-income countries. AIDS research and treatment 2011: 749041.

4. World Health Organization HIV/AIDS Programme, UNAIDS, UNICEF (2010) Towards Universal Access: Scaling up priority HIV/AIDS interventions in the health sector. . Geneva: World Health Organization.

5. World Health Organization (2010) Antiretroviral therapy for HIV infection in adults and adolescents – recommendations for a public health approach. Available: http://whqlibdoc.who.int/publications/2010/9789241599764_eng.pdf. Accessed 2011 May 12.

6. Marston M, Todd J, Glynn JR, Nelson KE, Rangsin R, et al. (2007) Estimating 'net' HIV-related mortality and the importance of background mortality rates. AIDS 21 Suppl 6: S65-71.

7. Todd J, Glynn JR, Marston M, Lutalo T, Biraro S, et al. (2007) Time from HIV seroconversion to death: a collaborative analysis of eight studies in six low and middle-income countries before highly active antiretroviral therapy. AIDS 21 Suppl 6: S55-63.

8. Morgan D, Mahe C, Mayanja B, Okongo JM, Lubega R, et al. (2002) HIV-1 infection in rural Africa: is there a difference in median time to AIDS and survival compared with that in industrialized countries? AIDS 16: 597-603.

9. Stover J, Bollinger L, Walker N, Monasch R (2007) Resource needs to support orphans and vulnerable children in sub-Saharan Africa. Health Policy Plan 22: 21-27.

10. Lofland JH, Locklear JC, Frick KD (2001) Different approaches to valuing the lost productivity of patients with migraine. Pharmacoeconomics 19: 917-925.

11. Jeffries K, Siphanmbe H, Kinghorn A (2006) The economic impact of HIV/AIDS in Botswana. Econsult Pty Ltd.

12. Koopmanschap MA, van Ineveld BM (1992) Towards a new approach for estimating indirect costs of disease. Soc Sci Med 34: 1005-1010.

13. Johannesson M, Karlsson G (1997) The friction cost method: a comment. J Health Econ 16: 249-255; discussion 257-249.

14. Flessa S, Marschall P (2009) Socio-economic impact of antiviral intervention. Handb Exp Pharmacol: 347-374.

15. UNAIDS (2009) AIDS epidemic update : November 2009. Geneva: UNAIDS.

16. Haacker M (2004) HIV/AIDS: The Impact on the Social Fabric and the Economy. In: Haacker M, editor. The Macroeconomics of HIV/AIDS. Geneva: International Monetary Fund.

17. Booysen FlR, Arntz T (2003) The methodology of HIV/AIDS impact studies: a review of current practices. Social Science & Medicine 56: 2391-2405.

18. King JT, Jr., Justice AC, Roberts MS, Chang CC, Fusco JS (2003) Long-term HIV/AIDS survival estimation in the highly active antiretroviral therapy era. Med Decis Making 23: 9-20.

19. World Health Organization HIV/AIDS Programme (2009) Rapid advice: Antiretroviral therapy for HIV infection in adults and adolescents. Geneva. Available: http://www.who.int/hiv/pub/arv/rapid_advice_art.pdf. Accessed 2011 Mar 31.

20. Habyarimana J, Mbakile B, Pop-Eleches C (2007) HIV/AIDS, ARV Treatment and Worker Absenteeism: Evidence from a Large African Firm.

21. Larson BA, Fox MP, Rosen S, Bii M, Sigei C, et al. (2008) Early effects of antiretroviral therapy on work performance: preliminary results from a cohort study of Kenyan agricultural workers. AIDS 22: 421-425.

22. Thirumurthy H, Saravanan R, Srinivas G, Jafri A, Sreevidya J, et al. (2008) The impact of antiretroviral therapy on socioeconoic outcomes of HIV-infected patients in Tamil Nadu family care continuum program, India. . XVII International AIDS Conference. Mexico City, Mexico.

23. Fox MP, McCoy K, Larson BA, Rosen S, Bii M, et al. (2010) Improvements in physical wellbeing over the first two years on antiretroviral therapy in western Kenya. AIDS Care 22: 137-145.

24. Fox MP, Rosen S, MacLeod WB, Wasunna M, Bii M, et al. (2004) The impact of HIV/AIDS on labour productivity in Kenya. Trop Med Int Health 9: 318-324.

25. Larson BA, Fox MP, Rosen S, Bii M, Sigei C, et al. (2009) Do the socioeconomic impacts of antiretroviral therapy vary by gender? A longitudinal study of Kenyan agricultural worker employment outcomes. BMC Public Health 9: 240.

26. Ajithkumar K, Iype T, Arun KJ, Ajitha BK, Aveenlal KP, et al. (2007) Impact of antiretroviral therapy on vocational rehabilitation. AIDS Care 19: 1310-1312.

27. Morineau G, Vun MC, Barennes H, Wolf RC, Song N, et al. (2009) Survival and quality of life among HIV-positive people on antiretroviral therapy in Cambodia. AIDS Patient Care STDS 23: 669-677.

28. Sgombich X, Bahamondes L, Cid C (2006) Quality of life in work, absenteeism, and HAART in workers living with HIV. XVI International AIDS Conference. Toronto, Canada.

29. Eholie S, Nolan M (2003) Antiretroviral treatment can be cost-savings for industry and life-saving for workers: a case study from Cote d'Ivoire's private sector. In: Moatti J, Barnett T, Coriat B, Souteyrand Y, Dumoulin J et al., editors. Economics of AIDS and access to HIV/AIDS care in developing countries: issues and challenges. Paris, France: Agence Nationale de Recherches sur le Sida. pp. 329-346.

30. Muirhead D, Kumaranayake L, Hongoro C, Charalambous S, Grant A, et al. (2006) Health care costs, savings, and productivity benefits resulting from a large employer sponsored ART program in South Africa. XVI International AIDS Conference. Toronto, Caanada.

31. Rosen S, Ketlhapile M, Sanne I, Desilva MB (2008) Differences in normal activities, job performance and symptom prevalence between patients not yet on antiretroviral therapy and patients initiating therapy in South Africa. AIDS 22 Suppl 1: S131-139.

32. World Bank (2009) World Development Indicators Online 8 Oct 2009 ed: World Bank.

33. Beegle K, DeWeerdt J, Dercon S (2007) Orphanhood, and the long-run impact on children. Center for the studies of African economics. Available: http://www.csae.ox.ac.uk/workingpapers/pdfs/2007-08text.pdf. Accessed 2011 Apr 15.

34. Pridmore P (2008) Access to conventional schooling for children and young people affected by HIV and AIDS in sub-Saharan Africa: a cross-national review of recent research evidence. London: SOPHIE. 34 p.

35. The Global Fund to Fight AIDS Tuberculosis and Malaria (2010) The Global Fund Results report 2010: innovation and impact. Geneva.

36. The Global Fund to Fight AIDS Tuberculosis and Malaria (2010) Financial and health impacts of continued support to the three diseases: long-term estimates. . Geneva: The Global Fund to Fight AIDS Tuberculosis and Malaria.

37. Thirumurthy H, J G-Z, M G (2008) The economic impact of AIDS treatment: labor supply in Western Kenya. Journal of Human Resources 43: 511-552.

38. Coetzee D, Boulle A, Hildebrand K, Asselman V, Van Cutsem G, et al. (2004) Promoting adherence to antiretroviral therapy: the experience from a primary care setting in Khayelitsha, South Africa. AIDS 18 Suppl 3: S27-31.

39. Linnemayr S, Lawson BS, Glick P, Wagner G (2011) Economic Status and Coping Mechanisms of Individuals Seeking HIV Care in Uganda. Journal of African Economies 20: 505-529.
